# Diet-Induced Regulation of Bitter Taste Receptor Subtypes in the Mouse Gastrointestinal Tract

**DOI:** 10.1371/journal.pone.0107732

**Published:** 2014-09-19

**Authors:** Gaia Vegezzi, Laura Anselmi, Jennifer Huynh, Elisabetta Barocelli, Enrique Rozengurt, Helen Raybould, Catia Sternini

**Affiliations:** 1 CURE Digestive Diseases Research Center, Digestive Diseases Division, Department of Medicine, David Geffen School of Medicine, University of California Los Angeles, Los Angeles, California, United States of America; 2 Department of Neurobiology, David Geffen School of Medicine, University of California Los Angeles, Los Angeles, California, United States of America; 3 Veterans Administration, Greater Los Angeles Health system, Los Angeles, California, United States of America; 4 Department of Pharmacy, University of Parma, Parma, Italy; 5 Department of Anatomy, Physiology, & Cell Biology, School of Veterinary Medicine, University of California Davis, Davis, California, United States of America; German Institute of Human Nutrition Potsdam-Rehbrücke, Germany

## Abstract

Bitter taste receptors and signaling molecules, which detect bitter taste in the mouth, are expressed in the gut mucosa. In this study, we tested whether two distinct bitter taste receptors, the bitter taste receptor 138 (T2R138), selectively activated by isothiocyanates, and the broadly tuned bitter taste receptor 108 (T2R108) are regulated by luminal content. Quantitative RT-PCR analysis showed that T2R138 transcript is more abundant in the colon than the small intestine and lowest in the stomach, whereas T2R108 mRNA is more abundant in the stomach compared to the intestine. Both transcripts in the stomach were markedly reduced by fasting and restored to normal levels after 4 hours re-feeding. A cholesterol-lowering diet, mimicking a diet naturally low in cholesterol and rich in bitter substances, increased T2R138 transcript, but not T2R108, in duodenum and jejunum, and not in ileum and colon. Long-term ingestion of high-fat diet increased T2R138 RNA, but not T2R108, in the colon. Similarly, α-gustducin, a bitter taste receptor signaling molecule, was reduced by fasting in the stomach and increased by lowering cholesterol in the small intestine and by high-fat diet in the colon. These data show that both short and long term changes in the luminal contents alter expression of bitter taste receptors and associated signaling molecules in the mucosa, supporting the proposed role of bitter taste receptors in luminal chemosensing in the gastrointestinal tract. Bitter taste receptors might serve as regulatory and defensive mechanism to control gut function and food intake and protect the body from the luminal environment.

## Introduction

The sense of taste is important to evaluate the quality of nutrients and distinguish between safe and dangerous food prior to ingestion [1], [2]. Sweet, umami and bitter tastes are detected by G protein- coupled receptors, known as taste receptors (TRs), which interact with specific G protein subunits, including α-gustducin and α-transducin, and other transducers such as phospholipase Cβ2 and transient receptor potential channel type (TRPM) 5, to activate effector systems leading to intracellular Ca^2+^ increase and transmitter release [3]–[6]. Sweet and umami are typically associated with palatability, thus inducing acceptance behavior and initiating physiological responses that facilitate digestion and absorption. By contrast, bitter taste likely acts as a warning mechanism against toxic or harmful chemicals, which are often bitter, thus evoking signals to induce avoidance or rejection [7], [8]. Bitter taste receptors (T2Rs) are found in a variety of species from avian and amphybians to mammals [9]. There are more than 30 T2Rs in rodents and about 25 in humans, some of which have homologs in mouse, though the nomenclature differs. T2Rs can be broadly tuned, being activated by a variety of bitter substances that can be structurally divergent, whereas others are more discretely or narrowly tuned by recognizing a few or single compounds [10]. Expression of sweet/umami (T1R) and bitter (T2R) taste receptors and their signaling molecules has been reported in several extra-oral sites including the digestive [11]–[16], respiratory [17]–[22] and reproductive systems [23], [24], and the brain [25] strongly supporting the concept that there is more than a “taste” function for taste receptors and that they exert non-gustatory functions outside the mouth, which might vary according to the site of expression [4], [26].

The gastrointestinal (GI) tract is the largest interface between our body and the environment, and the lining of the GI tract is continuously exposed to environmental factors, including nutrients, microorganisms and toxins [27]–[32]. Molecular sensing by GI epithelial cells plays a critical role in the control of multiple fundamental functions in digestion and also activates hormonal and/or neural pathways leading to the regulation of caloric intake, pancreatic insulin secretion, GI motility and secretion, and metabolism. Molecular sensing in the GI mucosa is also responsible for the detection of ingested harmful drugs and toxins, thereby initiating responses critical for survival. Enteroendocrine cells throughout the GI tract serve as specialized transducers of luminal factors by releasing signaling molecules that in turn activate nerve fibers and local or distant targets to initiate appropriate physiological responses [27], [28], [30]–[32]. The discovery that taste receptors, including T2Rs and related molecule transcripts, are expressed in the GI mucosa and that taste signaling molecules are localized to enteroendocrine cells [12], [13] has provided support to the concept that the GI tract detects luminal stimuli through utilization of taste-related molecules that in turn modulate GI functions. This idea is further supported by observations that bitter tastants induce an increase in intracellular Ca^2+^ and release of GI peptides in enteroendocrine cell lines, and that bitter tastants evoke prostaglandin-regulated anion secretion in the colon *in vivo* [14], [33]–[35]. Additional support for this hypothesis derives from studies showing that intraluminal administration of T2R ligands induces activation of vagal afferent neurons in the nucleus of the solitary tract, which are known to affect GI functions, and changes in behavior including inhibition of food intake, avoidance, and delay of gastric emptying [36]–[38]. Taste receptors might act as the initial molecular mechanisms activating enteroendocrine cells to release peptides in response to luminal stimuli, with T2Rs also serving as putative defense tools to reduce or eliminate potential toxic substances. It is, therefore, of major importance to elucidate in detail the regional and cellular distribution of individual T2Rs in different regions of the gut.

The aim of this study was to test the hypothesis that T2Rs in different regions of the mouse GI tract are regulated by feeding, fasting and diet manipulations. We focused on two distinct mouse T2Rs: mT2R138, which is selectivity tuned for the detection of thiourea/isothiocyanates such as phenylthiocarbamide (PTC) and propylthiouracil (PROP), and mT2R108, which is broadly tuned and detects denatonium benzoate (DB) [4], [14], [33]–[35]. Transcripts for both these receptors have been detected in the gut mucosa and enteroendocrine cell lines [14], [15] and, when stimulated by intraluminal agonists, activate distinct vagal afferent neurons [38]. We tested whether mRNA expression of T2R138, T2R108 and their major signaling molecule, α-gustducin is affected by feeding and fasting, since bitter tastants, including PTC and DB, have been shown to affect food intake and induce aversion [36], [37], [39]. We have used a cholesterol-lowering diet, which has been reported to regulate T2Rs gene expression via the cholesterol-sensitive transcription factor SREB-2 [34]. We have also used a high fat, high caloric diet (45% or 60% fat, 4.7–5 kcal/g) which induces obesity [40] and is known to change gut microbiota with increase in the proportion of Gram-negative bacteria and to induce chronic inflammation [41]–[44]. Our study showed a differential level of expression of these T2Rs throughout the gut mucosa and changes in their expression in distinct regions of the gut in response to fasting and different types of feeding supporting our hypothesis that intraluminal contents regulate T2Rs expression and the concept that T2Rs have distinct roles depending on the gut region and diet manipulation.

## Materials and Methods

### Animals and Diets

Experiments were performed in 4-week-old male C57/BL6 mice (Charles River Laboratory International, Inc, Hollister, CA). Controls and experimental groups were of comparable age.

### Ethics Statement

Animal care and procedures were in accordance with the National Institutes of Health recommendations for the humane use of animals. Experimental procedures were reviewed and approved by the Animal Research Committee of the University of California, Los Angeles and by the Institutional Animal Care and Use Committee (UC Davis, CA). All efforts were made to minimize the number of animals used and their suffering.

### Feeding Manipulations

For fasting and re-feeding experiments, mice were fasted 18 hours (n = 11) and re-fed for 4 hours (n = 10) after fasting; control mice received regular chow (n = 11). For cholesterol lowering diet, mice were fed regular chow (n = 12) or chow supplemented with Lovastatin (100 mg/100 g chow) and Ezetimibe (21 mg/100 g chow) (LE) for 7 days (n = 16). For high fat diet (HF), mice were fed 45% (Research Diets, D12451) or 60% (Research Diets, D12492) fat by calories diet (4.7–5 kcal/g) for 2 or 8 weeks, whereas control mice were fed a 10% (Research Diets, D12450B, 3.8 kcal/g) fat by calories diet (n = 10–15 per group). The characteristics of mice fed HF diets have been described in detail [40]. Briefly, both HF-diets induced significant increase in body weight, adiposity and inflammatory markers compared to mice fed with a 10% fat diet [40]. At the end of the period on different diets, mice were euthanized by isoflurane overdose for tissue removal. The whole gastrointestinal tract was removed and the lumen was flushed with saline. Specimens of the stomach, small intestine (duodenum, jejunum, ileum) and colon (proximal and distal) were snap frozen for qRT-PCR [45] or fixed for 2 h in 4% paraformaldehyde in 0.1 M phosphate buffer, pH 7.4 (PFA) followed by 25% sucrose in 0.1 M phosphate buffer for 12–24 hours at 4°C for immunohistochemical analysis [46], [47].

### RNA Extraction and Quantitative Real-Time RT-PCR

**Figure 1:**
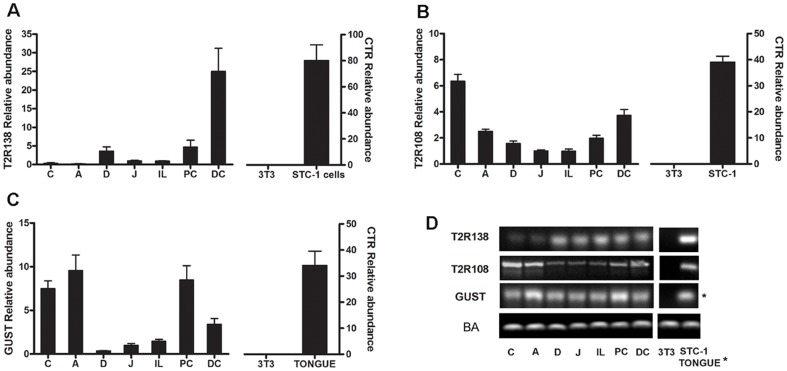
Expression of mT2R138, mT2R108 and α-Gustducin (Gust) mRNA in the mouse Gastrointestinal Tract. mRNA levels were analyzed in the different regions of the GI tract with qRT-PCR and normalized to β-actin levels in each tissue (A–C). Relative quantities were determined using the comparative ΔΔCt method. Each cDNA sample was amplified in duplicate and all data are expressed as the mean ± S.E.M. T2R138 expression was very low in the stomach compared to the small and large intestine (A), whereas T2R108 is more abundant in the stomach compared to the intestine (B). α-Gustducin is more abundant in the stomach and colon compared to the other regions (C). D: Single bands of the predicted size were found for each primer, in all GI segments analyzed as well as in STC1 cells or the tongue, which served as positive controls. No signal was detected with any of the primer in 3T3 cells, which were used as negative control. A:antrum, C:corpus, D:duodenum, J: jejunum, I: ileum, PC: proximal colon, DC: distal colon, STC1: STC 1 cells, BA: β actin; CTR, control; * the tongue is shown as control for α-gustducin.

Total RNA was isolated from mouse gastrointestinal tissue (stomach, duodenum, jejunum, ileum, proximal and distal colon) using Absolutely RNA Miniprep Kit (Stratagene, La Jolla, CA). Treatment with DNase I from this kit was performed at 37°C for 15 minutes to degrade any genomic DNA contamination. Spectrophotometric analysis of the sample consistently showed absorption ratio (OD) OD_260nm_/OD_280nm_>1.8, indicating excellent purity of the ribonucleic acids. We used 2% agarose gel electrophoresis to assess genomic DNA contamination and RNA integrity, which was verified by the presence of two distinct bands that correspond to 18S and 28S rRNA (Fig. S1). Only samples in which two distinct bands were detected and without genomic DNA contamination were used for RT-PCR analysis. Complementary DNA was synthesized using Superscript III Reverse Transcriptase kit (Invitrogen) according to the manufacturer's instructions on a DNA Thermal Cycler Engine, BIO-RAD. Quantitative real-time reverse transcription polymerase chain reactions (qRT-PCR) were performed using Taqman Gene expression assays for T2R138 (Applied Biosystem, Mm01700131_s1), T2R108 (Applied Biosystem Mm00498514_s1), α-gustducin (Applied Biosystem, Mm01165313_m1) and HMG-CoA reductase (3-hydroxy-3-methyl-glutaryl-CoA reductase) (Applied Biosystem, Mm01282492_m1). HMG-CoA reductase is the rate-controlling enzyme of the metabolic pathway producing cholesterol and is increased when cholesterol is low [34]. HMG-CoA reductase was measured to verify the effectiveness of pharmacological treatment in cholesterol lowering diet Standard thermal cycles for Taqman Gene assays consisting of one cycle of 50°C for 2 min and 95°C for 10 min, followed by 40 cycles of 95°C for 15 sec (denaturation) and 60°C for 1 min (annealing and elongation) were run on a Mx3000P Real-time PCR Detection System (Stratagene). Data were collected in real time and analyzed with Mx Pro 1000 software. For all primers, an enteroendocrine cell line (STC-1) expressing T2Rs and α-gustducin, and a fibroblast cell line (3T3) not expressing T2Rs or α-gustducin [14] were used as a positive and negative control, respectively. Samples were run at least in duplicates in separate experiments; no-RT (Fig. S2) and distilled RNAse-free water controls were always included. qRT-PCR products were checked by 4% agarose gel electrophoresis for bands of correct sizes (see Fig. 1). The relative abundance of each mRNA and the relative changes in mRNA expression in different experimental conditions were calculated using the Delta Delta cycle threshold (Ct) method (ΔΔCT) as previously described [45], [48], where ΔCT is equal to the difference between the target Ct and the reference Ct (C_T_ target gene − C_T_ reference gene), ΔΔCT =  ΔC_T_ sample – ΔC_T_ calibrator (control), and RQ =  Relative quantification  = 2^−ΔΔCT^ that represents fold changes compared to calibrator or control. ß actin (Applied Biosystem Mm01205647_g1) and 18S RNA (Applied Biosystem Mm03928990_g1) were initially used as reference genes. Both ß actin and 18S mRNA levels were comparable in different segments of the gastrointestinal tract and the relative expression of each target mRNA was similar when using ß actin or 18S (Figs. S3 and S4), thus we selected to use ß actin as reference gene for subsequent experiments with diet manipulations. The levels of ß actin mRNA were not affected by fasting and re-feeding, by low cholesterol or high fat diets (Fig. S5), confirming the suitability of this reference gene. Data were expressed relatively to an internal standard (jejunum) for the distribution of each target gene throughout the gastrointestinal tract; the jejunum was arbitrarily chosen as the unity of measurement and adjusted so that it had a mean relative mRNA level of 1. For diet manipulations, the control group for each experimental condition was set as internal standard. Control groups for fasting and re-feeding experiments and for cholesterol lowering diet were mice fed regular chow, whereas controls for high fat diet received 10% fat diet. All assays were validated for linearity of amplification efficiency. Since T2R138 levels are very low in the stomach, we performed all the previous controls at a higher number of cycles, which we used to detect C_T_ values between 40 and 50. Positive linear correlation, consistency of data for multiple replicates, correct base pair size and the observation that C_T_ values were undetectable in all negative controls ensured specificity of results in our experimental conditions for T2R138 expression.

### Immunohistochemistry

Immunohistochemistry was performed to characterize the cellular sites of expression of T2R138 in comparison to the distribution of α-gustducin [11]–[13], [15]. We could not determine the cellular sites of expression of T2R108 due to the lack of specific antibodies raised against this receptor for immunohistochemistry. Tissue was fixed in 4% paraformaldehyde in 0.1 M phosphate-buffered (pH 7.4) (PB) for 2 hours followed by 25% sucrose for 12–24 hours at 4°C [46], [47]. Immunohistochemistry was performed on 10 µm-thick frozen sections as described [49]. Briefly, sections were washed with 0.1 M PB, pretreated with 5% normal donkey serum in 0.5% Triton X-100/PB to block non specific binding and incubated overnight at 4°C with goat anti mouse T2R138 (Sc-34357, Santa Cruz Biotechnology) at 1∶250 dilution, rabbit anti mouse α-gustducin at 1∶250 (Sc-395, Santa Cruz Biotechnology), or rabbit anti Chromogranin A 1∶500 (Sc-1488; Santa Cruz Biotechnology) in 0.5% Triton X-100/PB. Chromogranin A was used as a general marker for enteroendocrine cells [50]. Tissues were then incubated in affinity-purified donkey anti-rabbit or anti goat ALEXA Fluor 488 (1∶1000; Invitrogen Molecular Probes, Eugene, OR) or Rhodamine Red X 568 (1∶300, Invitrogen Molecular Probes) for 2 hours at room temperature. Specificity controls included omission of the primary antibody and immunoblocking experiments. For the latter experiments, T2R138 and α-gustducin antibodies were pre-absorbed with the corresponding peptides (100 µg peptide in 0.5 ml PB) as recommended by the supplier (Santa Cruz Biotechnology) overnight, then used for immunofluorescence in parallel with sections incubated with the antibodies without pre-absorption. Specificity controls for double labeling immunofluorescence were performed to exclude non-specific binding due to the mixing of the antibodies or binding of the secondary antisera to the inappropriate primary antibodies [51]. Immunoreactivity was analyzed with a Zeiss 510 META laser scanning confocal microscope with a 63X PlanApo 1.4 numerical aperture objective (Carl Zeiss, Inc., Thornwood, NY). Images were adjusted for brightness and contrast using Adobe Photoshop 7.0 (Adobe System, Mountain View, CA).

### Statistic Analysis

Values were expressed as the mean ± S.E.M. One-way ANOVA followed by Bonferroni post-test for multiple comparisons or Student's t-test were used for statistical analysis for distribution and fasting/re-feeding data. Two-way ANOVA was used for statistical analysis of the difference in mRNA levels in groups where the results of one treatment were assessed on different GI specimens (LE and HF diet). P values <0.05 were taken as significant. The statistical software package Prism 5.0 (GraphPad Software, San Diego, CA) was used for these analyses.

## Results

### T2R138, T2R108 and α-Gustducin mRNA Expression in the Mouse GI Tract

qRT-PCR and gel electrophoresis showed the presence of the amplified products generated by the Taqman Gene Expression Assay primers specific for T2R138, T2R108, α-gustducin and β-actin in the different specimens of the GI tract (Fig. 1). T2R138 mRNA levels were more abundant in the distal colon, compared to the small intestine, with very low levels in the stomach (Fig. 1). By contrast, T2R108 mRNA expression was more abundant in the stomach, followed by colon and small intestine. α-gustducin transcript was also distributed throughout the GI tract with the highest levels in the stomach and colon. All quantifications are relative to an internal control, the jejunum, arbitrarily chosen, whose value was adjusted so that its mean relative mRNA level was 1. As shown in Fig. 1, T2Rs and α-gustducin mRNAs were not detected in 3T3 cells, a fibroblast cell line used as a negative control, and were abundant in STC-1 cells, a mouse enteroendocrine cell line, which has been well characterized for the expression of T2Rs or in mouse tongue, both used as positive controls [14], [15].

**Figure 2:**
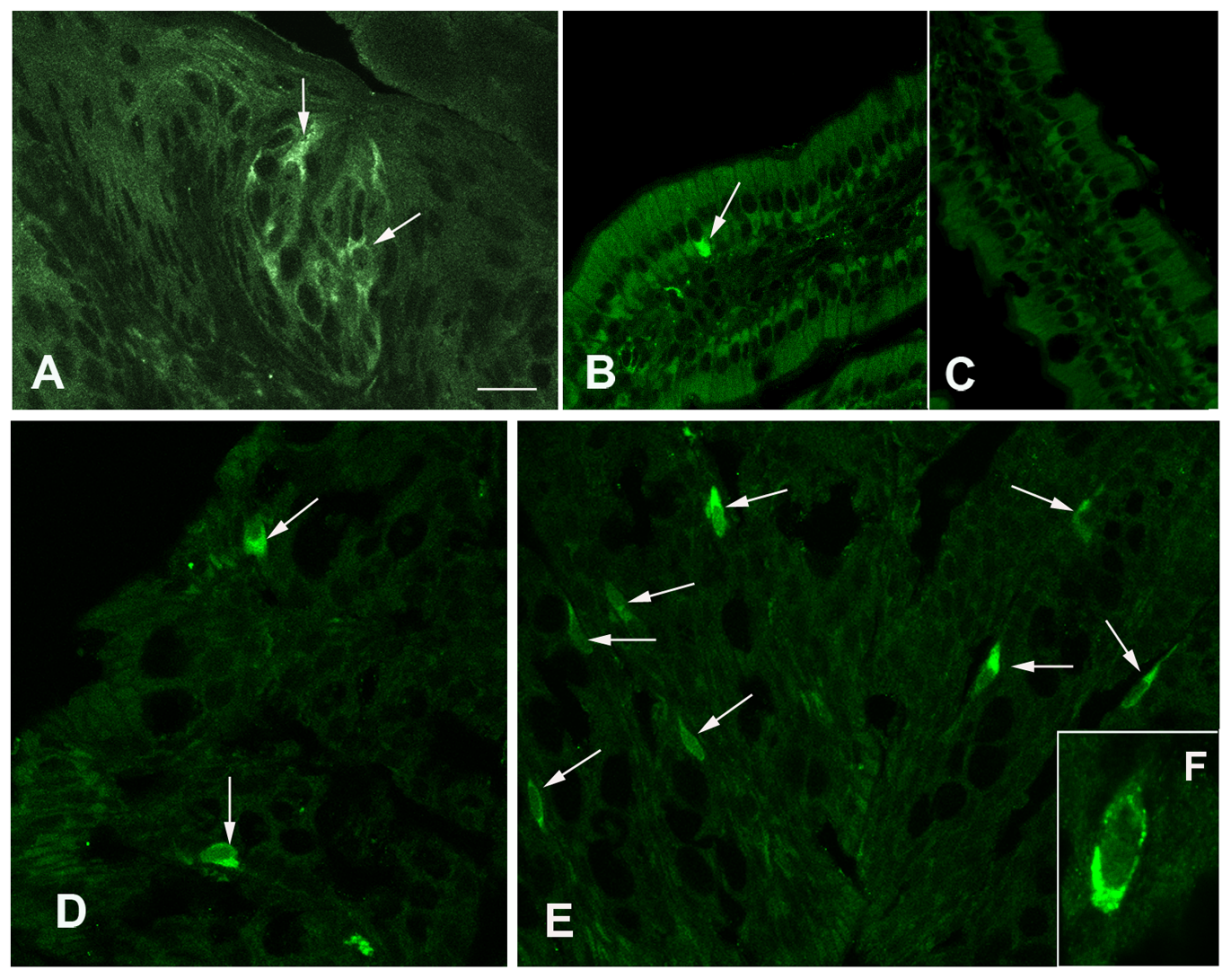
Distribution of T2R138 Immunoreactivity in the Gastrointestinal Tract. Confocal images. T2R138 immunostaining in cells (arrows) of the tongue (A) and in isolated cells (arrows) along the GI tract (B, D, E). B: T2R138 immunoreactive cell in the jejunum. C: Lack of specific staining in a section incubated with T2R138 antibody pre-adsorbed with the antigen against which the antibody was raised. D and E: T2R138 immunoreactive cells in the proximal (D) and distal (E) colon. F: high magnification of a T2R138 immunoreactive cell of the colon showing the granular staining concentrated toward the base of the cell. Calibration bar: 20 µm in A–E, 10 µm in F.

**Figure 3:**
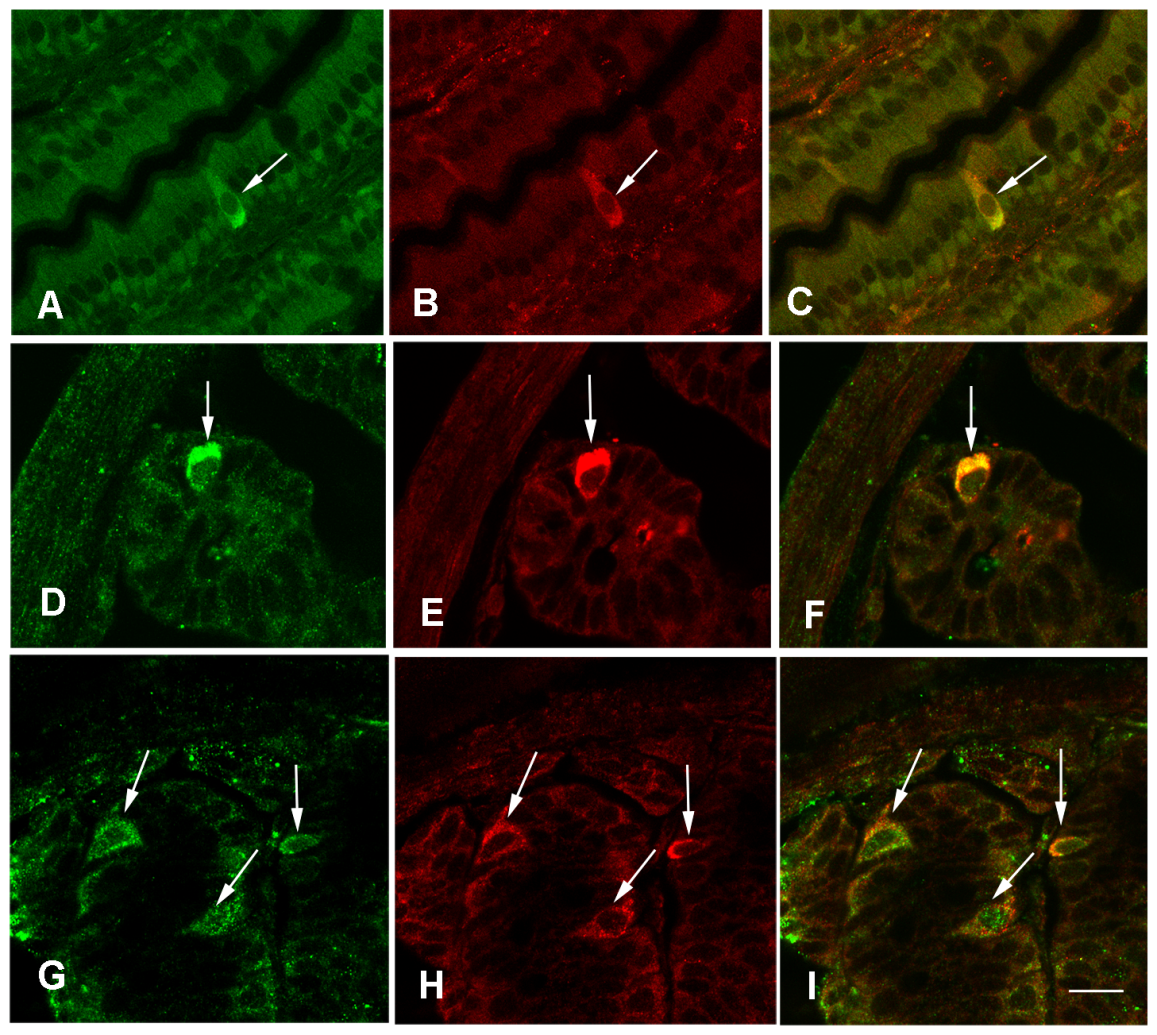
Cells Expressing T2R138 in the Gut. Confocal images of mouse ileum showing colocalization of T2R138 (green) (A) with α-Gustducin (red) (B) immunoreactivity; C: shows overlay of both T2R138 and α-Gustducin immunoreactivity. D–I: Confocal images showing colocalization of T2R138 (green) (D, G) with chromogranin A, a marker of enteroendocrine cells (red) (E, H) and overlay of both immunoreactivities in the same cells (F,I) in the ileum (D–F) and distal colon (G–I). Calibration bar: 20 µm.

Immunohistochemistry showed that T2R138 immunoreactivity was localized to isolated epithelial cells distributed throughout the mouse GI tract (Fig. 2), which were more abundant in the colon compared to other GI regions. Specificity of immunoreaction was demonstrated by the strong labeling of taste bud cells in the tongue (Fig 2A) and immunoblocking experiments showing abolition of T2R138 (Fig 2C) or α-gustducin (not shown;[12], [15]) immunostaining when antibodies were pre-incubated with an excess of the appropriate peptide. T2R138 immunoreactivity was localized to most cells immunoreactive for α-gustducin (Fig. 3), confirming that the same cells contain the receptor and its signaling protein, though the overlapping was not complete. α-gustducin immunoreactive cells were distributed throughout the GI mucosa (not shown) confirming previous observations in the mouse and human GI mucosa [11]–[14]. Double labeling with chromogranin, a well established marker for endocrine cells [50] showed that most T2R138 immunoreactive cells contained chromogranin A immunoreactivity (Fig. 3) confirming that T2R138, as α-gustducin, is expressed in enteroendocrine cells [12], [13], [15].

### Effect of Fasting/Re-feeding and Different Diets on T2R138, T2R108 and α-Gustducin Expression

**Figure 4:**
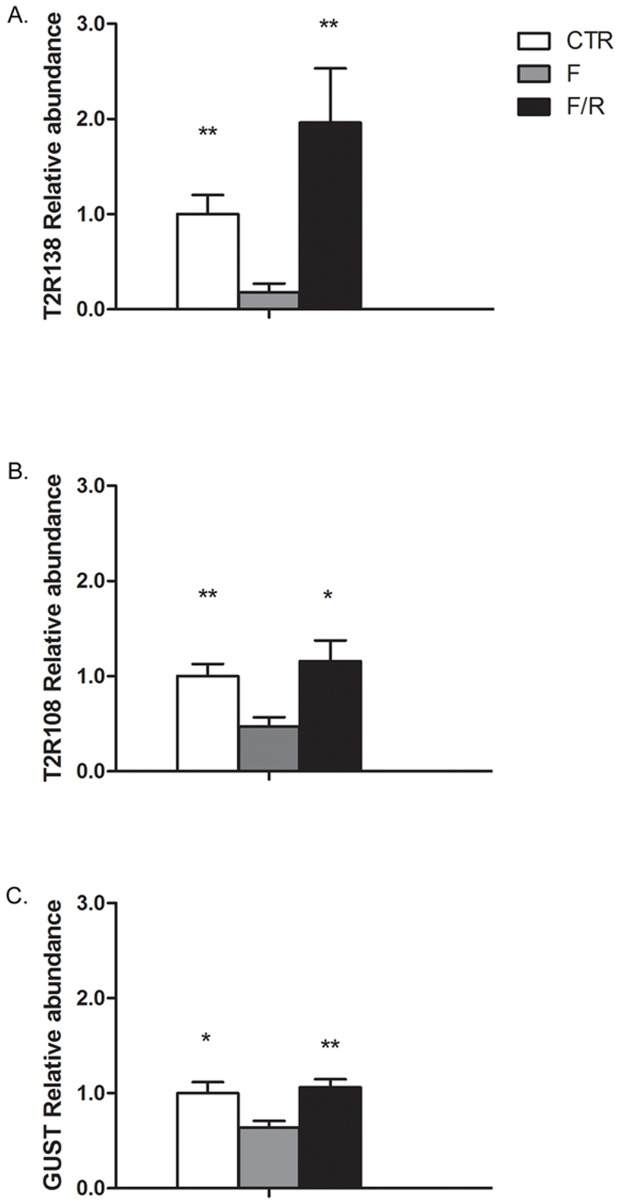
TR2138, T2R108 and α-Gustducin (Gust) Regulation by Fasting/Re-feeding in the Stomach. mRNA levels for each transcript were analyzed by qRT-PCR and normalized to β-actin. T2R138, T2R108 and α-gustducin mRNA levels were markedly decreased by fasting (82%, 53% and 37%, respectively compared to controls) and restored by re-feeding. *p<0.05, **p<0.01 vs. fasted. CTR, control; F, fasted; F/R, re-feeding following fasting.

Following 18 h fasting, there was a pronounced decrease in T2R138, T2R108 and α-gustducin transcripts in the stomach vs. control (82%, 53% and 37%, respectively, p<0.05 vs. controls). All transcripts were restored to control levels 4 hours after re-feeding (Fig. 4). There was also a marked decrease of α-gustducin mRNA in the duodenum with restoration to control values after re-feeding (p<0.01). There were no detectable changes in T2R138, T2R108 or α-gustducin mRNA levels in response to fasting in the other regions of the gut (not shown).

**Figure 5:**
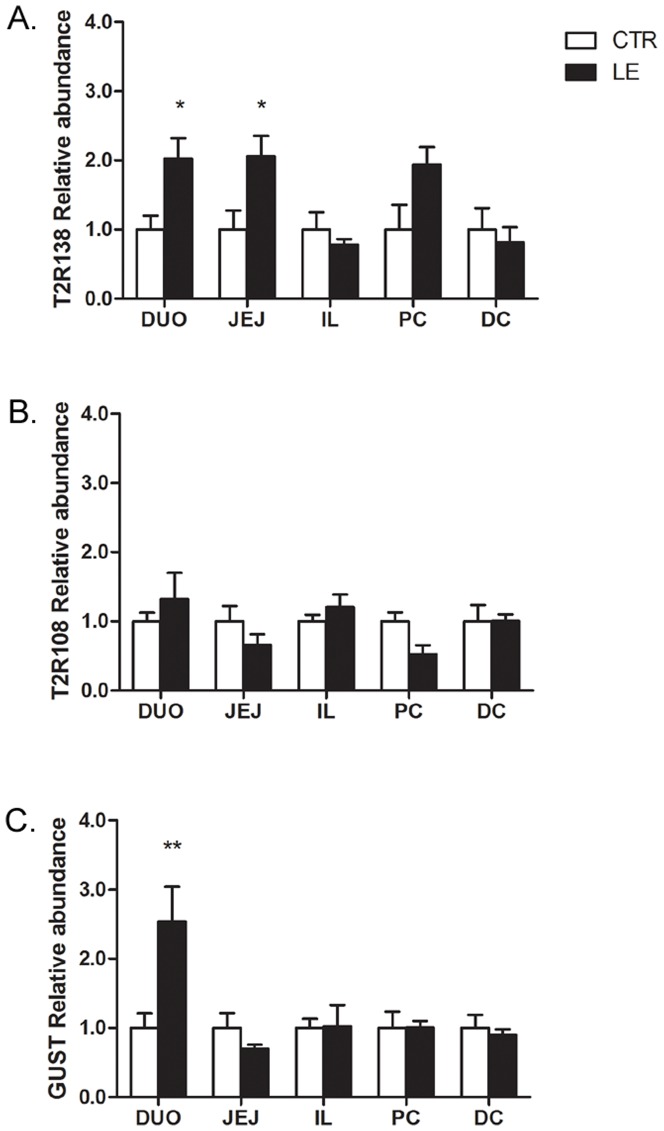
Effect of a Low Cholesterol (LE) Diet on T2R138, T2R108 and α-Gustducin (Gust) Expression. qRT-PCR analysis shows that T2R138 mRNA is significantly up-regulated in the duodenum (DUO) and jejunum (JEJ), but not the ileum (IL), proximal colon (PC) and distal colon (DC), following 7 days of a cholesterol lowering diet. α-gustducin mRNA is also significantly increased in the duodenum. *p<0.05, **p<0.01 vs. control. By contrast, T2R108 mRNA levels were not affected by this diet in any regions of the gut.

In animals fed normal chow diet supplemented with Lovastatin and Ezetimibe to lower cholesterol absorption, there was a significant increase in T2R138 mRNA expression in the duodenum and jejunum, but not in other regions of the small or large intestine (Fig. 5), compared to control chow diet. α-gustducin was also up-regulated by low cholesterol diet in the duodenum (Fig. 5). By contrast, T2R108 mRNA levels were not significantly modified in any GI regions (Fig. 5). To verify the effectiveness of pharmacological treatment in cholesterol lowering diet, we checked the expression of HMG-CoA reductase. Since Lovastatin and ezetimibe reduce dietary cholesterol absorption, mice treated with these drugs respond as they are cholesterol starved resulting in a significant increase in HMG-CoA. In animals treated with Lovastatin and Ezetimibe, HMG-CoA reductase mRNA was significantly increased (4.07±0.79 in treated mice vs. 1.00±0.30 in controls, p<0.01; Fig. S6), an indication of sterol depletion confirming the cholesterol lowering effect of the treatment [34].

**Figure 6:**
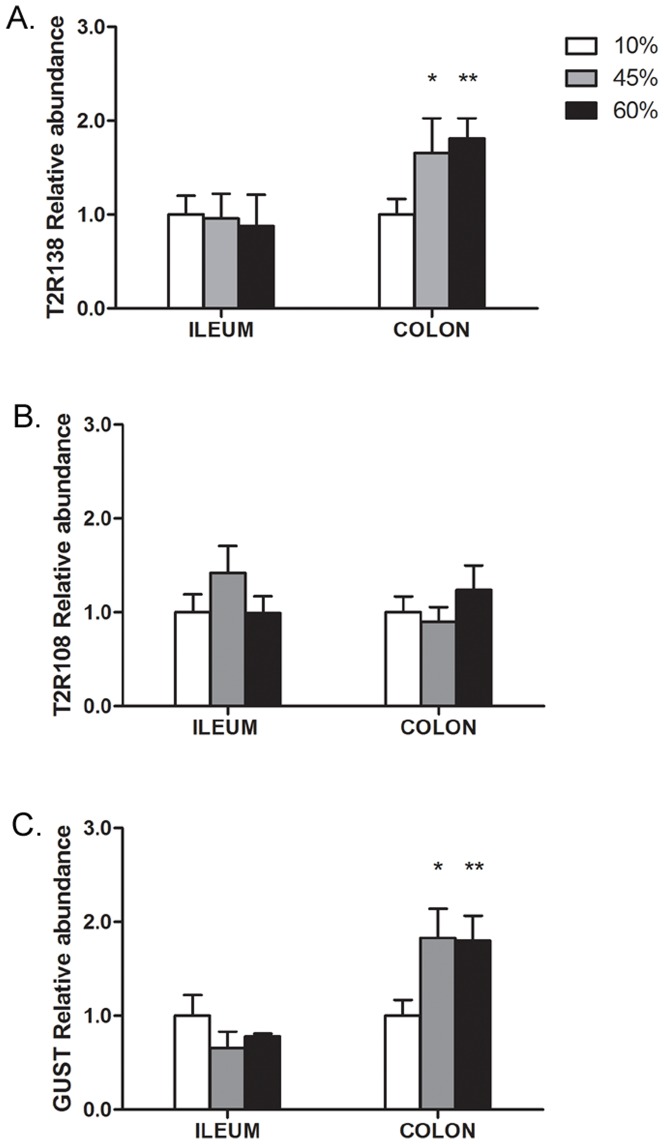
Effect of a High Fat Diet on T2R138, T2R108 and α-Gustducin (Gust) Expression in the Ileum and Colon. qRT-PCR analysis shows that T2R138 mRNA and α-gustducin mRNA levels are significantly (*p<0.05; **p<0.01) up-regulated in the colon, but not ileum by a long term (8 weeks) high fat (45% and 60%) diet compared to low (10%) fat diet. By contrast, T2R108 mRNA was not affected by this diet.

There was a significant increase in the levels of T2R138 mRNA and α-gustducin mRNA in the colon of mice that were fed a high fat diet (45% or 60% high fat) for 8 weeks, but not in the ileum, whereas no differences were observed in T2R108 expression in either the ileum or colon (Fig. 6). However, the same high fat diet did not induce any change in any of the transcripts analyzed when given for 2 weeks only (not shown), supporting the concept that the up-regulation of T2R138 and α-gustducin were more likely a consequence of the intraluminal changes induced by the high fat diet, than a direct effect of the diet itself.

## Discussion

This study shows that T2R138 and T2R108 transcripts are distributed throughout the mouse GI tract with different levels of expression that are affected by changes in the luminal content and provides evidence for the regional distribution of T2R138 in enteroendocrine cells throughout the GI tract with highest abundance in the colon. The vast majority of T2R138 positive cells contained chromogranin A, a marker for enteroendocrine cells and many of them also contain α-gustducin immunoreactivity. Given the vast co-localization of T2R138 with α-gustducin, which is expressed in distinct types of enteroendocrine cells, including CCK, GLP-1, PYY, ghrelin and 5-HT cells [12], [13], [15], [39], [52], it is presumed that T2R138 is also found in different populations of enteroendocrine cells. This conclusion is supported by findings of colocalization of immunoreactivity for human T2R38 (which is activated by PTC and PROP as mouse T2R138) with markers of different subpopulations of enteroendocrine cells including CCK, GLP-1, PYY in human large intestine (unpublished). We cannot exclude that some cells expressing T2R138 immunoreactivity are not enteroendocrine, since the colocalization between T2R138 and chromogranin immunoreactivity was not complete. These non-endocrine cells could include brush cells as suggested by previous studies showing immunoreactivity for α-gustducin, the major taste-receptor signaling molecule, in this type of cells [11]. However, we have not observed specific staining in enterocytes. By contrast, other T2Rs such as T2R131 have been localized to goblet cells in the colon [53]. Whether T2R108 is also expressed by enteroendocrine cells cannot be established at this time because of the lack of antibodies specific for this receptor.

Enteroendocrine cells are the primary chemoreceptors in the GI lumen and respond to luminal content by releasing chemical messengers that in turn activate neuronal pathways as well as local or distant targets to induce appropriate functional responses depending upon the initial stimulus [28], [30]–[32]. Since taste receptors act as sensors for nutrients and non-nutrients in the oral cavity, the presence of these receptors and their signaling molecules in enteroendocrine cells supports the concept that taste-related molecules participate in chemosensing processes responsible for the induction of functional responses to intraluminal contents. This notion is supported by the activation of vagal afferent neurons of the solitary tract innervating the gut by intraluminal T2R ligands including PTC and DB that activate T2R138 and T2R108, respectively [37], [38]. This prompted us to investigate whether changes in the luminal content modify the expression of T2R138 and T2R108, and of α-gustducin. Fasting has been shown to alter the levels of different hormones including a reduction in gastrin and an increase in somatostatin and ghrelin [39], [54], [55]. Gastrin and ghrelin cells contain taste-related molecules (Sternini, unpublished; [39], [52]) and a fasting-induced reduction of cells expressing taste signaling molecule and ghrelin, restored with re-feeding, has been reported in the pig stomach [52]. Gastrin exerts a variety of effects including stimulation of gastric secretion and mucosal protection, and ghrelin is an orexigenic and prokinetic hormone that regulates energy balance [56], [57]. The reduced expression of taste-related molecules could represent the initial mechanism controlling changes in gastrin and ghrelin release during fasting. The inhibition of gastrin results in reduction of secretion and motility, prolonging satiety and reducing food intake. By contrast, release of ghrelin will stimulate appetite, increase food intake and accelerate gastric emptying. With re-feeding, both taste-related molecules and gastric hormones are back to normal. The reduction of T2Rs and α-gustducin mRNAs by fasting in the stomach with normal levels being restored by re-feeding suggests that taste-related molecules serve as a mechanism for the modulation and maintenance of hormones balance by direct or indirect effect, which in turn might regulate food intake and gastric function. Indeed, activation of T2R138 and T2R108 with intraluminal ligands induces an increase in blood levels of ghrelin with early increase followed by late decrease in food intake and delaying in gastric emptying through a mechanism involving α-gustducin, since these effects were reduced in mice with α-gustducin genetic deletion [39].

T2Rs have also been hypothesized to serve as defense mechanisms to protect against harmful substances through the release of peptides/hormones that alter GI function to limit absorption of and contact with toxins or poisons. This is in agreement with the conditioned flavor avoidance and reduction of food intake and gastric emptying delays induced by administration of T2R ligands [36], [37], [39]. The up-regulation of T2Rs, including T2R138, together with the enhanced PTC-induced CCK and GLP-1 release in endocrine cell lines and in the small intestine in response to a drug-induced sterol depletion mimicking a naturally low cholesterol diet is also consistent with this idea [34]. CCK regulates postprandial gastric emptying and secretion, as well as protein and fat digestion, and GLP1 slows gastric emptying and inhibits intestinal motility [58], [59]. Our study defined the specific regions of mouse gut where T2R138 transcripts are up-regulated by a sterol depletion diet, which include the duodenum and jejunum, but not the ileum, proximal and distal colon. In this context, it is of interest that naturally low cholesterol diets are rich in plants that often contain bitter, potentially toxic components, compared to a high-cholesterol diet containing mostly animal meat. Activation of T2Rs expression might function to prevent the consumption and absorption of potentially toxic/bitter substances in plant-derived foods.

Recent studies in the respiratory system have provided strong evidence that T2R38 in human and T2R138 and T2R108 in mice are activated by pathogen-derived quorum sensing molecules in the upper respiratory tract and nasal mucosa and trigger an innate immune response and antimicrobial effect to prevent bacteria infection [20]–[22]. We hypothesized that T2Rs, specifically T2R138, which responds to isothiocyanates as the human T2R38, in the colon has a similar function as in the respiratory system to detect bacterial molecules, since T2R138 is most abundant in the distal colon, a region particularly rich in bacteria. The human intestine hosts about 100 trillion microorganisms, representing hundreds of different species, and the density of bacteria in the colon has been estimated at 10^11^ to 10^12^ cells per milliliter, which makes the large intestine one of the most densely populated microbial habitats on Earth [60]. Our study shows a significant increase in the expression of T2R138 and α-gustducin in the colon, but not the small intestine, in mice that have been fed a high fat diet (45% and 60% fat) vs. a control, low fat diet (10% fat) for 8 weeks. There is increasing evidence that long-term high fat diet causes changes in the composition and quantity of the gut microflora [41], [61], [62], which is associated with a chronic low grade inflammation and is likely to represent the initial stage of obesity and metabolic disorders [42], [44]. The level of expression of T2R138 was not affected by the same high-fat diets administered for two weeks (not shown), suggesting that T2R138 up-regulation is not due to a direct effect of the high-fat diet on the epithelium but an indirect effect due to intraluminal changes induced by high-fat diets. T2R138 might serve as a defensive mechanism against pathogenic bacteria, perhaps initiating an inflammatory response to combat bacterial invasion or affecting mucosal barrier function.

Bitter tasting compounds are many and structurally diverse [10]. They belong to distinct chemical families, such as peptides, amino acids, fatty acids, alcohol, steroids, lactones and flavonoids [10] found in food and food-borne products, poisons, and drugs. Despite the large number of bitter taste receptors, ∼25–30 T2Rs in mammals [9], it is not surprising that many T2Rs are activated by numerous and various compounds given the vast array of tastants [10]. Furthermore, polymorphisms of some T2Rs, including T2R38, have been reported, which affect individual ability to perceive bitterness, which might influence nutrient intake and dietary preference and be associated with diet-related disorders [63], [64]. The ability of many T2Rs to respond to various ligands and the diverse distribution of these T2Rs ranging from taste buds to a variety of systems such as the respiratory, reproductive, gastrointestinal and nervous system, support the concept that distinct populations of T2Rs exert different functions depending upon the site of expression and ligand. Overall, the localization of T2R138 in enteroendocrine cells in different regions of the gut, the expression of α-gustducin in distinct types of enteroendocrine cells, including CCK, PYY, GLP1, ghrelin and 5-HT cells [12], [13], [15], [39] and the differential activation of T2R138, T2R108 and α-gustducin by diet manipulations, support the concept of a different role for T2Rs depending upon receptor subtype, region of the gut and interaction with multiple luminal components.

## Supporting Information

Figure S1 **Specificity controls for RNA integrity and genomic DNA contamination.** RNA quality was verified by the presence of two distinct bands corresponding to 28S and 18S rRNA on 2% agarose gel as shown in lanes 2–4, 6 and 7. By contrast, lane 5 is an example in which the two bands are less clearly demarcated and with RNA smearing below the two bands, whereas lane 1 is an example of RNA degradation resulting in the loss of the 28S rRNA band and an accumulation of degraded RNA near the bottom of the gel. The absence of genomic DNA (gDNA) contamination was confirmed by the lack of signal at the top of the gel, where genomic DNA should appear since it runs much slower through the gel matrix compared to RNA. Only extracts in which RNA integrity was confirmed by two distinct 28S and 18S bands (e.g. lanes 2–4,6,7) and without genomic DNA contamination were used to run qRT-PCR experiments.
(TIF)

Figure S2 **No-RT PCR controls for mouse colon and STC 1 cells.** In order to further verify the absence of genomic DNA, we included no-RT controls (PCR with no reverse transcriptase) in our experiments. Figure S2 illustrates a 2% agarose gel electrophoresis of qRT-PCR products obtained from mouse distal colon (DC, upper gel) and STC-1 cells (lower gel), for all the different primers. Odd numbered lanes: RT-PCR samples; even numbered lanes: No-RT PCR controls. In our standard operating conditions, target genes were undetectable in no-RT as well as in water controls (not shown), confirming that no DNA was found in samples or in reagents. BA, ß actin; T2R138, bitter taste receptor 138; T2R108, bitter taste receptor 108; GUST, α-gustducin; Std: standard 100 bp ladder.
(TIF)

Figure S3 **Relative T2R138 distribution along the mouse gastrointestinal tract using two distinct reference genes.** A and B compare the expression of T2R138 mRNA in different regions of the gastrointestinal tract using β actin (BA) RNA (A) or 18S RNA (B) as a reference gene. Results using the two different reference genes were comparable. C, gastric corpus; A: gastric antrum; D, duodenum; J, jejunum; I, ileum; PC, proximal colon; DC, distal colon.
(TIF)

Figure S4 **Distribution of β actin and 18S mRNA along the mouse gastrointestinal tract.** The levels of expression of the reference genes β actin (BA) and S18 mRNA do not changes significantly in the different regions of the gastrointestinal tract and are comparable to the levels in STC 1 cells (positive control) and 3T3 cells (negative control). C, gastric corpus; A: gastric antrum; D, duodenum; J, jejunum; I, ileum; PC, proximal colon; DC, distal colon.
(TIF)

Figure S5 **Expression of the reference gene, β actin in different regions of the mouse gastrointestinal tract in response to different diet manipulations.** β actin (BA) mRNA levels do not change in the mouse duodenum (A) from mice fed a lowering cholesterol (LE) diet compared to control mice (CTR), in the mouse large intestine (B) following high fat (HF, 45 and 60% vs. 10% low fat) diet, and in the mouse stomach (C) following fasting-re-feeding (F/R). BA expression is well conserved in different regions and in many experimental conditions, confirming the validity of BA as reference gene.
(TIF)

Figure S6 **HMG-CoA reductase mRNA in mice fed with lowering cholesterol diet.** HMG-CoA reductase mRNA expression was evaluated to verify the effectiveness of cholesterol lowering diet. HMG-CoA reductase mRNA was significantly increased (p<0.05) during the cholesterol lowering Lovastatin + Ezetimibe (L/E) diet compared to control (CTR) mice. The significant increase in HMG-CoA reductase mRNA confirms the cholesterol lowering effect of the treatment [34].
(TIF)
