# Supplementary figures and images for: Diet-Induced Regulation of Bitter Taste Receptor Subtypes in the Mouse Gastrointestinal Tract
Source: PLoS One. 2014 Sep 19;9(9):e107732. doi: 10.1371/journal.pone.0107732 (PMC4169553; doi:10.1371/journal.pone.0107732)

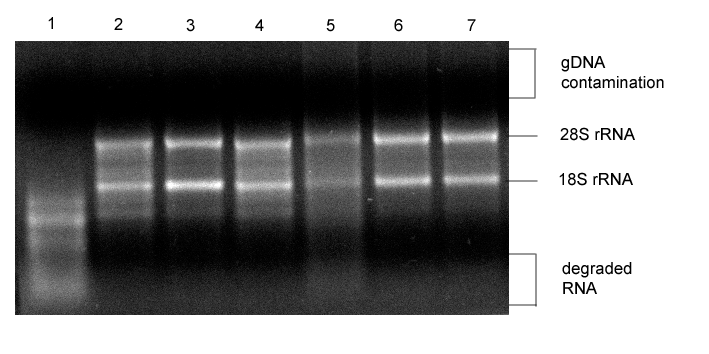

Supplement: Figure S1 — Specificity controls for RNA integrity and genomic DNA contamination. RNA quality was verified by the presence of two distinct bands corresponding to 28S and 18S rRNA on 2% agarose gel as shown in lanes 2–4, 6 and 7. By contrast, lane 5 is an example in which the two bands are less clearly demarcated and with RNA smearing below the two bands, whereas lane 1 is an example of RNA degradation resulting in the loss of the 28S rRNA band and an accumulation of degraded RNA near the bottom of the gel. The absence of genomic DNA (gDNA) contamination was confirmed by the lack of signal at the top of the gel, where genomic DNA should appear since it runs much slower through the gel matrix compared to RNA. Only extracts in which RNA integrity was confirmed by two distinct 28S and 18S bands (e.g. lanes 2–4,6,7) and without genomic DNA contamination were used to run qRT-PCR experiments. (TIF) [file pone.0107732.s001.tif]

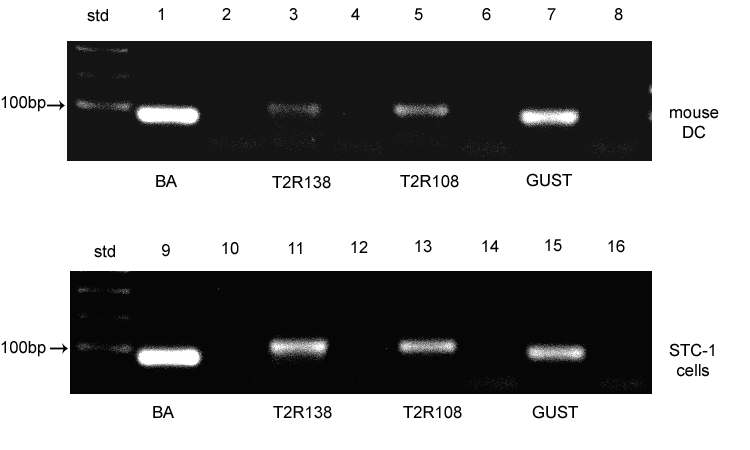

Supplement: Figure S2 — No-RT PCR controls for mouse colon and STC 1 cells. In order to further verify the absence of genomic DNA, we included no-RT controls (PCR with no reverse transcriptase) in our experiments. Figure S2 illustrates a 2% agarose gel electrophoresis of qRT-PCR products obtained from mouse distal colon (DC, upper gel) and STC-1 cells (lower gel), for all the different primers. Odd numbered lanes: RT-PCR samples; even numbered lanes: No-RT PCR controls. In our standard operating conditions, target genes were undetectable in no-RT as well as in water controls (not shown), confirming that no DNA was found in samples or in reagents. BA, ß actin; T2R138, bitter taste receptor 138; T2R108, bitter taste receptor 108; GUST, α-gustducin; Std: standard 100 bp ladder. (TIF) [file pone.0107732.s002.tif]

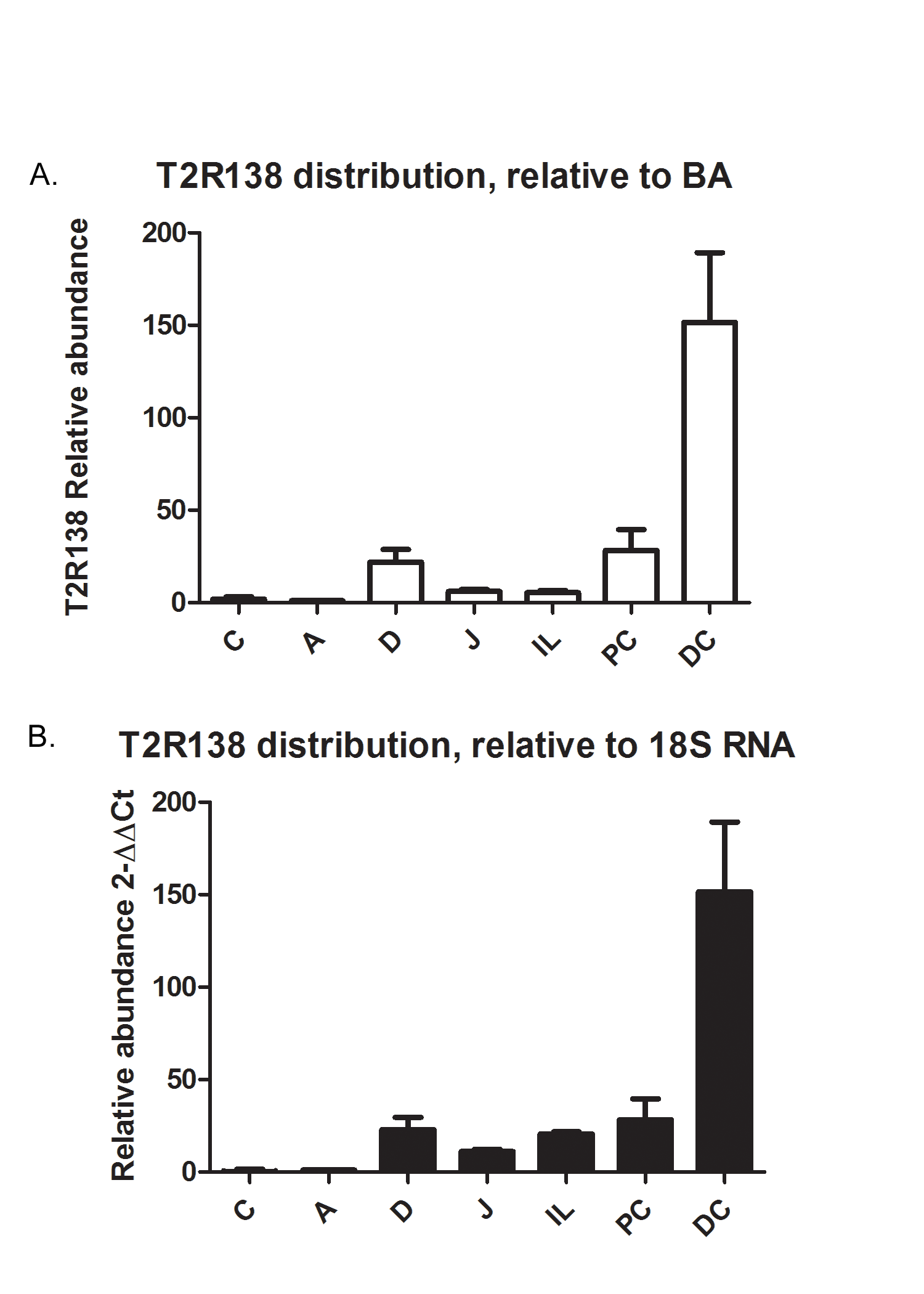

Supplement: Figure S3 — Relative T2R138 distribution along the mouse gastrointestinal tract using two distinct reference genes. A and B compare the expression of T2R138 mRNA in different regions of the gastrointestinal tract using β actin (BA) RNA (A) or 18S RNA (B) as a reference gene. Results using the two different reference genes were comparable. C, gastric corpus; A: gastric antrum; D, duodenum; J, jejunum; I, ileum; PC, proximal colon; DC, distal colon. (TIF) [file pone.0107732.s003.tif]

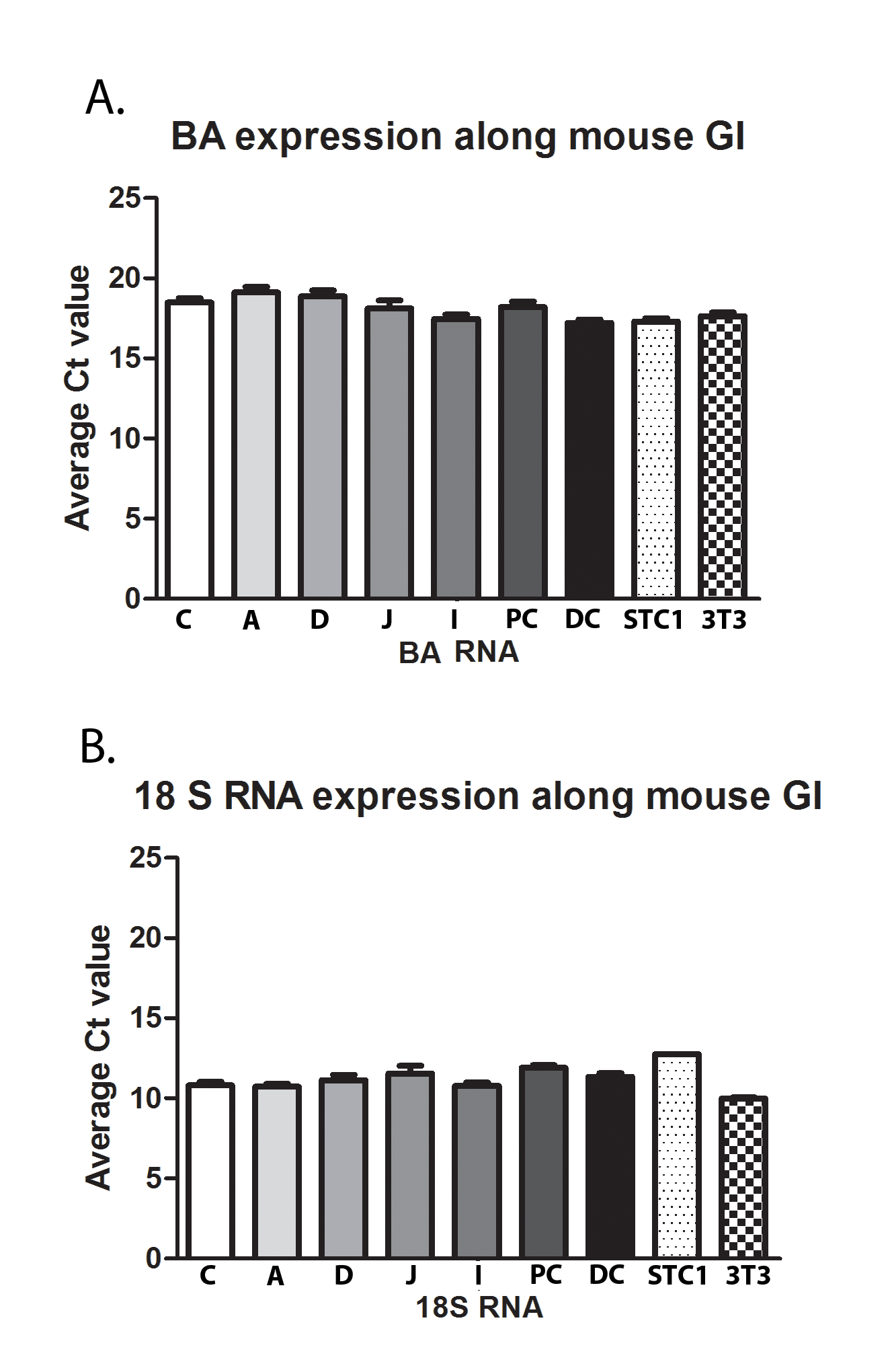

Supplement: Figure S4 — Distribution of β actin and 18S mRNA along the mouse gastrointestinal tract. The levels of expression of the reference genes β actin (BA) and S18 mRNA do not changes significantly in the different regions of the gastrointestinal tract and are comparable to the levels in STC 1 cells (positive control) and 3T3 cells (negative control). C, gastric corpus; A: gastric antrum; D, duodenum; J, jejunum; I, ileum; PC, proximal colon; DC, distal colon. (TIF) [file pone.0107732.s004.tif]

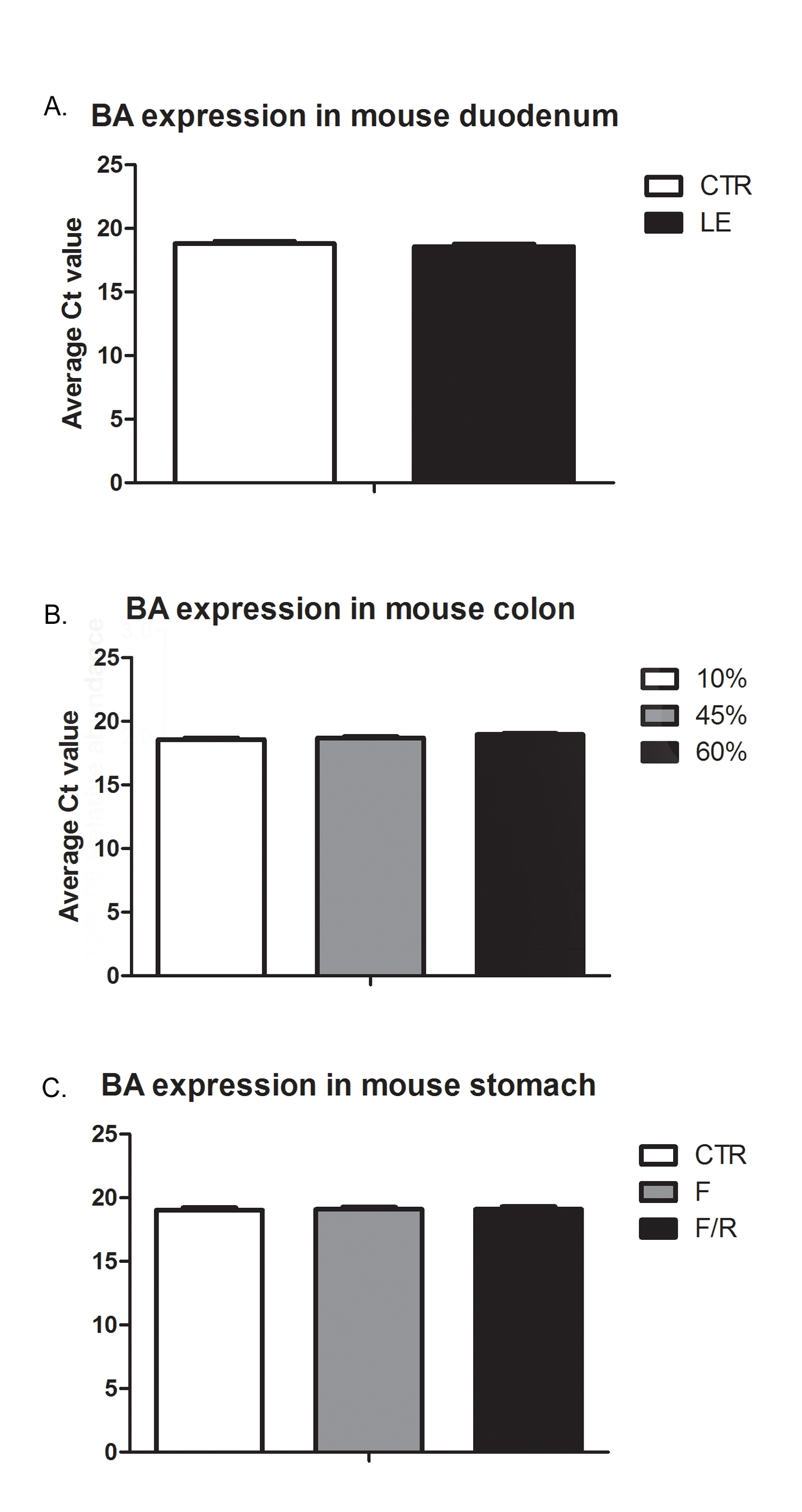

Supplement: Figure S5 — Expression of the reference gene, β actin in different regions of the mouse gastrointestinal tract in response to different diet manipulations. β actin (BA) mRNA levels do not change in the mouse duodenum (A) from mice fed a lowering cholesterol (LE) diet compared to control mice (CTR), in the mouse large intestine (B) following high fat (HF, 45 and 60% vs. 10% low fat) diet, and in the mouse stomach (C) following fasting-re-feeding (F/R). BA expression is well conserved in different regions and in many experimental conditions, confirming the validity of BA as reference gene. (TIF) [file pone.0107732.s005.tif]

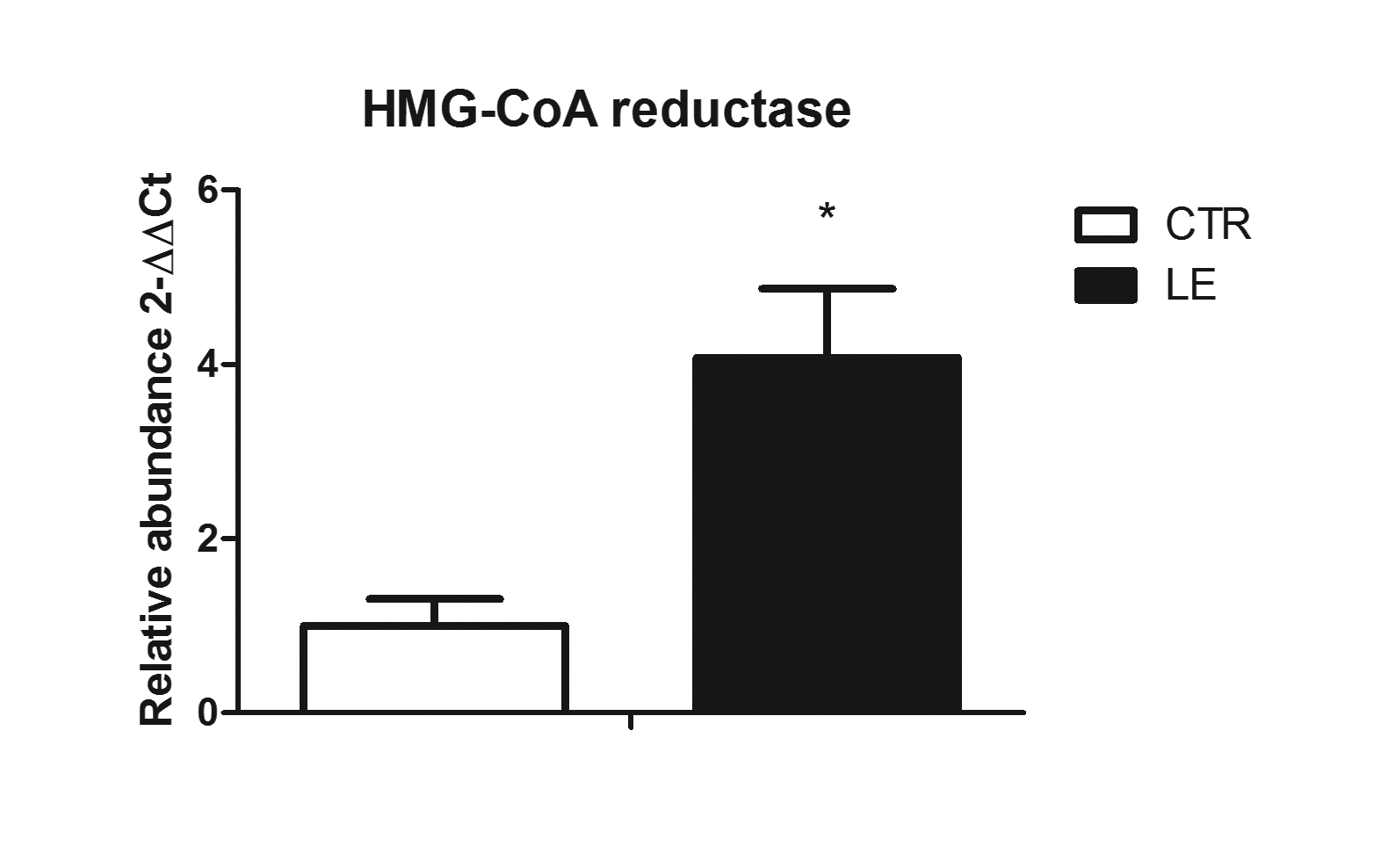

Supplement: Figure S6 — HMG-CoA reductase mRNA in mice fed with lowering cholesterol diet. HMG-CoA reductase mRNA expression was evaluated to verify the effectiveness of cholesterol lowering diet. HMG-CoA reductase mRNA was significantly increased (p<0.05) during the cholesterol lowering Lovastatin + Ezetimibe (L/E) diet compared to control (CTR) mice. The significant increase in HMG-CoA reductase mRNA confirms the cholesterol lowering effect of the treatment [34]. (TIF) [file pone.0107732.s006.tif]
